# Is adult separation anxiety associated with offspring risk for internalizing psychiatric problems?

**DOI:** 10.1017/S0033291721005249

**Published:** 2022-01-26

**Authors:** Megan C. Finsaas, Daniel N. Klein

**Affiliations:** 1 Department of Epidemiology, Columbia University, New York, NY, USA; 2 Department of Psychology, Stony Brook University, Stony Brook, NY, USA

**Keywords:** Adult separation anxiety, child separation anxiety, familial transmission, family study, offspring

## Abstract

**Background.:**

Awareness of adult separation anxiety (ASA) is growing, but there is a dearth of knowledge about how separation anxiety aggregates in families. We examined the intergenerational associations of separation anxiety and other forms of internalizing problems in an American community sample of 515 predominantly white children and their parents.

**Methods.:**

Children’s separation anxiety (CSA), depression, and other anxiety disorders were modeled as latent factors using diagnoses from interviews and symptom scores from questionnaires completed by mothers, fathers, and children when children were 9 years old and again 3 years later. Parents’ separation anxiety was assessed via a questionnaire and parents’ other anxiety, depressive, and substance use disorders were assessed with a diagnostic interview when children were nine. Relationships between parents’ and children’s psychopathology were modeled using s.e.m.

**Results.:**

Mothers’ and fathers’ ASA were related to all three psychopathology factors in offspring, over and above other parental disorders, in concurrent and prospective analyses. CSA was also related to maternal depression concurrently and prospectively and to maternal anxiety prospectively. Of all paternal psychopathology variables, only ASA was significantly related to children’s psychopathology in either model.

**Conclusions.:**

Results indicate that parental separation anxiety is an important, but non-specific, risk factor for children’s psychopathology. The pathway by which this risk is transmitted may be genetic or environmental, and the observed statistical associations likely also encompass child-to-parent effects.

## Introduction

It is well known that anxiety disorders run in families (Lawrence, Murayama, & Creswell, 2019; Merikangas, Avenevoli, Dierker, & Grillon, 1999). For childhood and adolescent anxiety, this aggregation reflects parent-to-child and child-to-parent effects (Ahmadzadeh et al., 2019; Knappe et al., 2009), as well as shared genetic liability (Boomsma, Van Beijsterveldt, & Hudziak, 2005). But the familial patterning of one particular anxiety disorder – separation anxiety – is less well characterized. This is because separation anxiety has not historically been recognized as a problem that occurs in adulthood, instead conceptualized and classified until recently as something that children experience in relation to their parents or primary caregivers (Bowlby, 1973). Consequently, studies on separation anxiety in parents, or adult separation anxiety (ASA), and in turn how separation anxiety clusters in families, are limited compared to other anxiety disorders.

The surprisingly high prevalence and clinical significance of ASA suggest that understanding the familial patterning of separation anxiety is important. Lifetime rates in the community are 7% (Shear, Jin, Ruscio, Walters, & Kessler, 2006) and rates in clinical samples are substantially higher (Pini et al., 2010; Silove, Marnane, Wagner, Manicavasagar, & Rees, 2010b). It predicts poorer treatment outcomes (Aaronson et al., 2008; Kirsten, Grenyer, Wagner, & Manicavasagar, 2008; Miniati et al., 2012) and is associated with functional impairment, even beyond the effects of comorbid disorders (Shear et al., 2006), and more so than other anxiety and mood disorders (Pini et al., 2010).

The only study on separation anxiety in families to date suggests that the association between ASA and children’s separation anxiety (CSA) is strong and highly specific. However, this study used a small sample of 54 parent–child dyads (Manicavasagar, Silove, Rapee, & Waters, 2001). We expand on this past work by examining the associations between parents’ and CSA and other internalizing psychopathology using a larger community sample and multiple assessment points. The timing of our assessments captures middle childhood and early adolescence, a period of high CSA (Copeland, Angold, Shanahan, & Costello, 2014), which also covers the median age of anxiety disorder onset (11 years; Kessler et al., 2005) and the beginning of the rise in rates of other internalizing disorders (Avenevoli, Knight, Kessler, & Merikangas, 2008). We also richly represent children’s psychopathology as latent variables that capture what is in common across multiple reporters (mothers, fathers, and children) and multiple methods (symptom reports and a semi-structured diagnostic interview).

Given that ASA, like all psychiatric problems, is highly comorbid with other disorders (Shear et al., 2006), we also test the specificity of ASA-CSA associations by adjusting for other forms of parental psychopathology, in contrast to the past study on familial transmission of separation anxiety, which only tested bivariate relationships (Manicavasagar et al., 2001). We also examine associations with multiple of children’s internalizing problems simultaneously in order to determine whether ASA is associated with children’s separation anxiety in particular or internalizing problems more generally. This is important because previous research suggests that disorders like depression and anxiety are better construed as specific manifestations of a higher-order internalizing factor and that familial transmission may occur at this level rather than at the level of specific disorders (Lahey, Van Hulle, Singh, Waldman, & Rathouz, 2011; Starr, Conway, & Brennan, 2014).

Moreover, because ASA is a newly-recognized clinical problem, it has not been well-validated as a clinical entity. According to Robins & Guze’s seminal work (1970), determining whether psychiatric disorders run in families is one of the five phases for establishing the validity of clinical problems. Family studies of psychiatric problems contribute to this aim by revealing meaningful patterns of associations across generations, regardless of the mechanisms of transmission. Thus, linking parents’ ASA to the better-established construct of CSA would increase the diagnostic validity of ASA.

In sum, we conducted a family study on the intergenerational patterning of separation anxiety in a predominantly white community sample from Long Island, NY in order to understand how ASA relates to CSA in particular and internalizing psychiatric problems more generally, and also to validate ASA.

## Methods

### Participants

The study sample is from an ongoing longitudinal study of children’s temperament and psychopathology; it includes 515 adult women and 510 adult men who completed a self-report measure of separation anxiety symptoms or a diagnostic interview, and 515 children who completed a diagnostic interview at one or two occasions. Two adult men had total ASA-27 scores which were over 8 s.d. above the mean (i.e. 79 and 81; range for the remainder of sample = 0–59), so they were excluded from analyses. Families with a 3-year-old child living within 20 miles of Stony Brook, New York, were eligible to participate in the larger study. Children with severe and disabling physical or developmental disorders were excluded. Participants were recruited from the community using commercial mailing lists (see Bufferd, Dougherty, Carlson, Rose, and Klein, 2012 for details). According to census data, the sample is demographically similar to the surrounding community. Parents provided written informed consent after receiving a description of the study. The study was approved by the human subjects review committee at Stony Brook University, and families were compensated. Families returned for assessments every 3 years. In the current study, we report on the wave 3 and wave 4 assessments.

### Measures

#### Adult separation anxiety

The Adult Separation Anxiety Questionnaire (ASA-27) is a 27-item self-report measure of separation anxiety symptoms experienced as an adult (over age 18) (Manicavasagar, Silove, Wagner, & Drobny, 2003). This measure was administered to parents at wave 3. Items are rated on a four-point scale (0 = *This has never happened*; 3 = *This happens very often*). The measure includes the same items as the Adult Separation Anxiety Semi-Structured Interview (ASA-SI) from the same group (Manicavasagar et al., 2003; Manicavasagar, Silove, & Curtis, 1997). Items come from clinical impressions, attachment theory, open-ended interviews, and versions of the DSM-IV separation anxiety criteria for youth. Case scores of ⩾16 or ⩾22 are recommended by the scale developers, the former for capturing all cases in the community and the latter being the intersection point between sensitivity and specificity. Using the latter cutoff and diagnoses from the ASA-SI as the criterion, sensitivity = 81%; specificity = 84%; misclassification rate = 17%; positive predictive power = 76%; and negative predictive power = 88% (Manicavasagar et al., 2003). The ASA-27 also had high internal consistency (Cronbach’s alpha = 0.95) and good test-retest reliability (*r* = 0.86, *p* < 0.001). Convergent validity of the ASA-27 is supported by an independent study that found that ASA-27 scores were strongly related to scores on the Structured Clinical Interview for Separation Anxiety Symptoms (SCI-SAS; *r* = 0.84), an interview that closely resembles questions on the structured interview used in the National Comorbidity Study-Replication (Cyranowski et al., 2002; Shear et al., 2006). The measure has shown partial strict measurement invariance across gender (Finsaas, Olino, Hawes, Mackin, & Klein, 2020). See Table 1 for Cronbach’s *α* and online Supplemental Table S1 for item descriptions and correspondence to DSM criteria.

#### Parent psychopathology

Adult participants were interviewed at waves 1 and 3 using the Structured Clinical Interview for DSM-IV, Non-Patient Version (SCID-NP; First, Gibbon, Spitzer, Williams, & Benjamin, 1997; Endicott & Spitzer, 1978). The SCID is considered the gold standard diagnostic interview for psychopathology in adults; for example, in the National Comorbidity Survey Replication Study, the validity of a different diagnostic interview was determined by comparison to the SCID-NP (Kessler et al., 2005). Masters-level clinicians and advanced clinical psychology graduate students conducted the interviews by telephone, which yield similar results as face-to-face interviews (Rohde, Lewinsohn, & Seeley, 1997). The wave 1 interview assessed lifetime diagnoses and the wave 3 interview the interval between the two assessments. Results from both interviews were combined to create lifetime diagnosis variables. When participants were unavailable, family history interviews were conducted with co-parents. To assess inter-rater reliability, second-raters independently rated audiotaped interviews. See Table 1 for kappas. Depressive disorder included major depressive disorder and dysthymia; anxiety disorder included panic disorder, specific phobia, social phobia, agoraphobia without panic disorder, GAD, obsessive compulsive disorder, and post-traumatic stress disorder; and substance use disorder included alcohol, cannabis, and hard drug abuse or dependence.

#### Children’s psychiatric diagnoses

The Kiddie Schedule for Affective Disorders and Schizophrenia Present and Lifetime Version (K-SADS-PL; Kaufman et al., 1997) was used to assess DSM-IV diagnoses in the children at the waves 3 and 4, when they were approximately 9 and 12 years old, respectively. The K-SADS-PL is widely used as the gold standard against which other diagnostic measures of psychopathology in youth are evaluated (e.g. Dougherty, Klein, and Olino, 2018; Kessler et al., 2009) and has been shown to generate valid diagnoses (Dougherty et al., 2018; Kaufman et al., 1997). Interviews were conducted separately with parents and children by advanced clinical psychology graduate students and a masters-level clinician and supervised by an experienced child psychiatrist and clinical psychologist. Further information was obtained to reconcile discrepancies as needed, and the interviewer made final ratings based on the combination of reports. To assess inter-rater reliability, second-raters independently rated video-tapes. See Table 1 for kappas. Depressive disorders included major depressive disorder, dysthymic disorder, depressive disorder NOS; other anxiety disorders included specific phobia, social phobia, generalized anxiety disorders, and panic/agoraphobia; and separation anxiety disorder was considered on its own. For the current project, the variables at wave 3 cover the child’s lifetime until that point (age 9) and the variables at wave 4 reflect whether a diagnosis was present at any point during the 3-year interval since wave 3.

#### Children’s anxiety symptoms

At waves 3 and 4, children and their parents completed the 41-item youth self-report and parent-report versions, respectively, of the Screen for Childhood Anxiety Related Disorders (SCARED; Birmaher et al. 1997, 1999). Children and their parents rated the presence of anxiety symptoms in the child over the past 3 months on a three-point scale. The SCARED is made up of five factor-analytically derived subscales: panic/somatic, general anxiety, separation anxiety, social phobia, and school phobia. These subscales reflect anxiety disorder symptoms as conceptualized in the DSM-IV. To create the indicators for the other anxiety factors, all subscales except for the separation anxiety scale were summed. This measure is widely used and its validity has been supported by numerous studies (Monga et al., 2000; Muris & Steerneman, 2001; Muris, Merckelbach, Van Brakel, & Mayer, 1999; Rappaport, Pagliaccio, Pine, Klein, & Jarcho, 2017). See Table 1 for Cronbach’s *α*.

#### Children’s depressive symptoms

At waves 3 and 4, children completed the 27-item youth self-report and parents completed the 17-item parent-report versions of the Children’s Depression Inventory (CDI; Kovacs, 1992). Children and their parents are asked to rate the presence of depressive symptoms in the youth in the past 2 weeks on a three-point scale. The CDI is widely used and has been shown to have concurrent, discriminant, and predictive validity (Dougherty et al., 2018; Timbremont, Braet, & Dreessen, 2004). See Table 1 for Cronbach’s *α*.

#### Data analysis

We computed descriptive statistics for demographic and study variables and conducted missingness analyses. To further characterize the sample, we estimated bivariate relationships between parent ASA scores at wave 3 and children’s diagnoses at waves 3 and 4 using point-biserial correlations. Next, to determine aggregation of separation anxiety in families, while also testing the specificity of associations with children’s psychopathology and addressing the issue of comorbidity in parents, we estimated models for mothers and fathers testing concurrent parent–child psychopathology relationships (parent and child variables measured at wave 3) and prospective parent–child psychopathology relationships (parent variables measured at wave 3 and child variables at wave 4). Children’s latent separation anxiety, other anxiety, and depression factors were indicated using the respective K-SADS diagnoses and mother, father, and child report of anxiety and depressive symptoms. K-SADS diagnoses were specified as categorical indicators. The residual variances of indicators by the same reporter were permitted to covary, as were the K-SADS indicators. Next, parents’ separation anxiety, measured by the ASA-27, and depressive, other anxiety, and substance use disorders, assessed by the SCID, were added as predictors. Models also included child ethnicity, gender, and age, and parent education, age, and marital relationship as covariates (child race was not included because empty cells with diagnosis variables caused problems with model convergence). Although cut-offs are somewhat arbitrary (Marsh, Hau, & Wen, 2004), current conventions suggest that adequate fit is indicated by CFI and TFI > 0.90, and RMSEA between 0.05 and 0.08 (Hu & Bentler, 1999; MacCallum, Browne, & Cai, 2006).

Descriptive statistics and correlations were computed in R Studio (version 1.2.1335; R Core Team, 2016) and CFA models were estimated in Mplus 8 (version 1.6; Muthen & Muthen, 2012–2018) using the robust weighted least squares estimator (WLSMV; Flora and Curran, 2004), which is suitable for categorical data, and maximum likelihood estimation on both the predictor and the outcome variables in order to utilize all available data (Enders, 2013).

## Results

At wave 3, children were 9.3 years old on average (s.d. = 0.42, range = 8.4–11.0); at wave 4, they were 12.7 years old on average (s.d. = 0.46, range = 11.5–14.2). About half were female (*n* = 224, 46.3%). Based on reports from parents about their children’s race, 457 children were white (88.7%), 42 were Black (8.2%), 14 were Asian (2.7%), and 1 was Native American (0.0%); 1 parent (0.0%) selected the ‘other’ category. Regarding ethnicity, 64 children were Hispanic (12.4%) according to the parent reports. At wave 3, mothers were 41.81 years old on average (s.d. = 4.83, range = 25.9–53.5) and fathers were 44.0 years old on average (s.d. = 5.85, range = 27.4–61.1). The majority of mothers (*n* = 413, 80.2%) and fathers (*n* = 411, 80.6%) were married to or living with the child’s biological parent. About half of the mothers (*n* = 276, 53.6%) and fathers (*n* = 216, 42.4%) had graduated from college with a 4-year degree. Data on demographic variables were self- and parent-reported on surveys (see online Supplemental Table S2 for description and coding of variables). Age was calculated using birthdate and date of assessment.

Mothers, fathers, and children included in the study sample were compared on all demographic variables to those who participated in the larger study but did not complete the aforementioned measures and/or interviews. Mothers and children did not differ. Fathers in the sample were more likely to be married and/or living with the child’s biological mother. See online Supplemental Table S3 for missingness rates and statistical comparisons and Table 2 for complete rates on study variables.

Descriptive statistics for children’s symptoms are in Table 2 descriptive statistics for children’s diagnoses are in Table 3. The average ASA score for mothers was 10.26 (s.d. = 9.37, range = 0–58) and the average for fathers was 8.10 (s.d. = 8.46, range = 0–54). Thirty-eight percent of mothers (193/514) and 19% of fathers (97/508) were diagnosed with a depressive disorder in their lifetimes, 38% of mothers (195/514) and 21% of fathers (109/506) were diagnosed with an anxiety disorder, and 22% of mothers (114/514) and 44% of fathers (224/508) were diagnosed with a substance use disorder. Generally, rates of psychiatric disorders are higher when based on cumulative prevalence across multiple assessments (Olino et al., 2012).

In bivariate analyses, maternal ASA was positively related to children’s lifetime separation anxiety assessed at wave 3, and both maternal and paternal ASA were positively related to children’s 3-year interval separation anxiety assessed at wave 4 (Table 3). Maternal ASA was also related to GAD at both waves and to lifetime depression at wave 3.

**Fig. 1.**
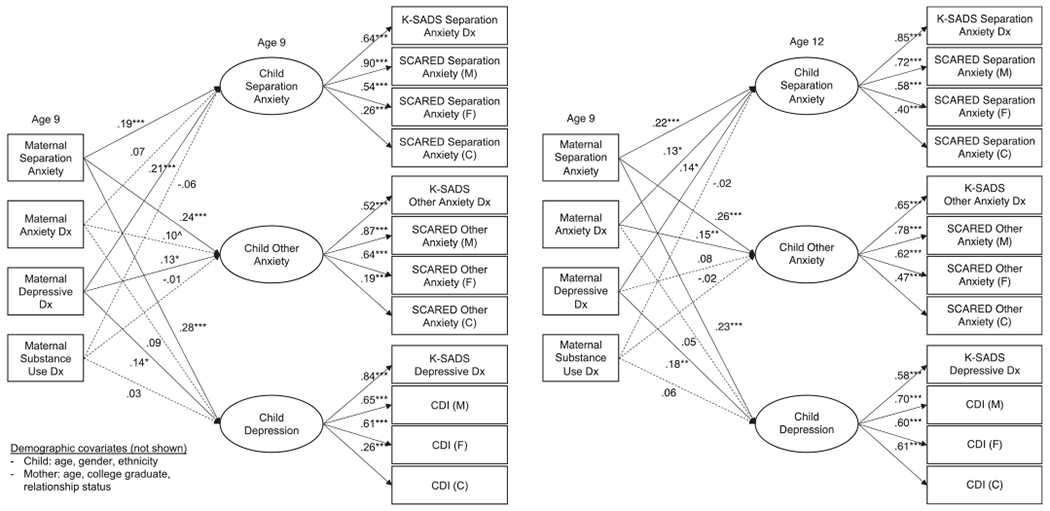
Concurrent and prospective relationships between maternal psychopathology and children’s separation anxiety, other anxiety, and depression factors. *Notes:* Solid lines indicate significant paths at *p* < 0.05. ***p < 0.001; **p < 0.01; *p < 0.05; ^*p* < 0.10. Dx, diagnosis; M, mother report; F, father report; C, child report; ASA-27, Adult Separation Anxiety Symptom Questionnaire; K-SADS, The Kiddie Schedule for Affective Disorders and Schizophrenia Present and Lifetime Version; SCARED, Screen for Child Anxiety Related Disorders; CDI, Children’s Depression Inventory. Maternal and child diagnoses at age 9 are from lifetime assessments. Child diagnoses at age 12 cover the interval since the last assessment. Covariances between independent variables are not shown, nor are covariances between children’s psychopathology factors, which were all significant at *p* < *0*.001. The residual variances of indicators by the same reporter were permitted to covary, as were the K-SADS indicators.

**Fig. 2.**
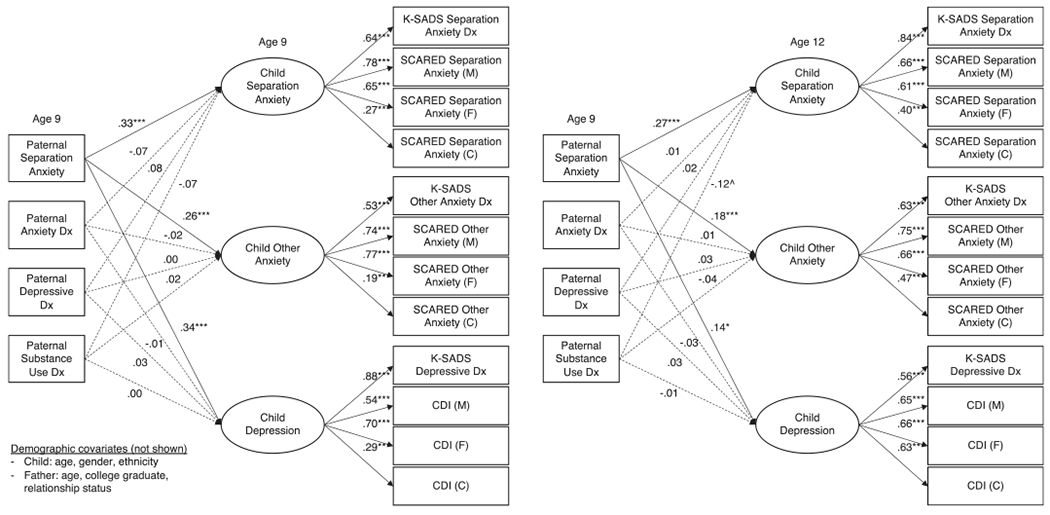
Concurrent and prospective relationships between paternal psychopathology and children’s separation anxiety, other anxiety, and depression factors. *Notes:* Solid lines indicate significant paths at *p* < 0.05. ***p < 0.001; **p < 0.01; *p < 0.05; ^*p* < 0.10. Dx, diagnosis; M, mother report; F, father report; C, child report; ASA-27, Adult Separation Anxiety Symptom Questionnaire; K-SADS, The Kiddie Schedule for Affective Disorders and Schizophrenia Present and Lifetime Version; SCARED, Screen for Child Anxiety Related Disorders; CDI, Children’s Depression Inventory. Paternal and child diagnoses at age 9 are from lifetime assessments. Child diagnoses at age 12 cover the interval since the last assessment. Covariances between independent variables are not shown, nor are covariances between children’s psychopathology factors, which were all significant at *p* < *0*.001. The residual variances of indicators by the same reporter were permitted to covary, as were the K-SADS indicators.

All four structural models fit reasonably well: CFI and RMSEA exceeded cutoffs for a good fit for all four models, and TFI exceeded the cutoff for 2 of the 4 (Table 4). All indicators loaded on respective factors at *p* < 0.001 (Figs 1 and 2). In all models, the children’s latent psychopathology factors were positively intercorrelated (separation anxiety/other anxiety: *β* = 0.61–0.68; separation anxiety/depression: *β* = 0.29–0.40; other anxiety/depression: *β* = 0.60–0.65).

Maternal ASA was non-specifically related to children’s internalizing psychopathology, beyond other maternal disorders, concurrently [*β* = 0.19–0.28) and prospectively (*β* = 0.22–0.26; Fig. 1). Maternal depression was likewise concurrently uniquely related to all three child factors (*β* = 0.13–0.21) and prospectively to CSA (*β* = 0.14) and children’s depression (*β* = 0.18). Maternal anxiety was not related to any child factors above and beyond other disorders concurrently but it was related to CSA (*β* = 0.13) and children’s other anxiety (*β* = 0.15) prospectively. Maternal substance use was not uniquely related to child internalizing psychopathology in either model. Like maternal ASA, paternal ASA was significantly related to the child psychopathology factors in the concurrent (*β* = 0.26–0.34) and prospective models (*β* = 0.14–0.27), beyond other paternal disorders (Fig. 2). In contrast to the maternal findings, however, none of the other paternal psychopathology variables was significantly associated with any of the child psychopathology factors.

## Discussion

Recent evidence suggests that separation anxiety is an impairing clinical problem that can occur during adulthood (Bögels, Knappe, & Clark, 2013). We examined the familial transmission of separation anxiety using a large community sample. Overall, our results highlight that maternal and paternal ASA is associated non-specifically with children’s internalizing psychopathology, including CSA, in middle childhood and early adolescence. We first showed how ASA was linked to children’s disorders in bivariate descriptive analyses. Maternal ASA was somewhat non-specifically related to children’s internalizing problems, whereas paternal ASA was not related to any of children’s psychopathology at age 9 and only to CSA between ages 9 and 12. The non-specificity of the associations for maternal ASA contradicts a study using a small clinical sample of children and their parents which found a high degree of specificity for ASA (Manicavasagar et al., 2001). The studies differ in terms of samples, but given the high rate of comorbid disorders in children in the clinical sample (54%), one would expect more rather than fewer associations between ASA and children’s other disorders. The differences may be due to the fact that their study combined mothers and fathers; if our samples were independent (i.e. mothers and fathers were not parents of the same children), combining mothers and fathers would likely weaken the multiple relationships observed with maternal ASA. Our study also had more power to detect associations based on larger sample size and a continuous measure of ASA.

We then explored whether ASA associations are specific to CSA or generalized to other forms of children’s internalizing disorders in structural equation models, and whether the associations between ASA and CSA are due to comorbid disorders in the parents. In addition to testing multiple relationships simultaneously, these models had the advantage over the bivariate tests of greater precision as children’s psychopathology was latent variables. These models are agnostic about the mechanism of transmission by which these associations arise and likely encompass environmental bidirectional parent-to-child and child-to-parent effects, as well as shared genetic liability. Concurrent models characterize familial clustering of separation anxiety and internalizing problems for youth in middle childhood when parent and child psychopathology are measured at the same time. The prospective models indicate the level of psychopathology we would expect to observe in 12-year-old children given the level of psychopathology in parents when children were 9 years old. Comparing associations between the two models facilitate inferences about the degree to which the patterning of relationships is stable across time and developmental periods.

Concurrent and prospective models in fathers and mothers show that ASA relates in a generalized, rather than specific, fashion to internalizing problems in youth. The associations are present when adjusting for the effects of maternal depressive, other anxiety, and substance disorders, suggesting they are not due to overlap with other forms of parental psychopathology; this highlights the significance of ASA in youth psychopathology, despite the generalized pattern of associations. Notably, the strength of the associations between ASA and children’s internalizing were similar in the concurrent and prospective models for mothers, suggesting they are stable from middle childhood to early adolescence, but somewhat attenuated in the prospective model for fathers.

Characterizing the associations with paternal ASA is in line with a call in a recent systematic review for continued research on ‘paternal mental health disorders, particularly anxiety disorders, and adolescent anxiety and depression’ (p. 244; Wickersham, Leightley, Archer, and Fear, 2020). The magnitudes of all ASA associations were larger for fathers compared to mothers in the concurrent models and for ASA-CSA in the prospective models. Paternal ASA was also the only paternal disorder with significant relationships with any child psychopathology factor. Given that women report higher levels of ASA compared to men (Aaronson et al., 2008; Silove et al., 2010b) and have higher rates of ASA disorder (Shear et al., 2006), it is possible that ASA in men reflects a particularly strong liability, which could place offspring at higher risk for poor outcomes. That is, the threshold for manifesting or disclosing symptoms may be higher for men compared to women due to genetic or environmental (e.g. reporter bias, stigma) factors, such that it requires more severe symptomatology for men to experience or endorse symptoms. However, in our sample, the ASA-27 demonstrated partial strict invariance across genders (Finsaas et al., 2020); this indicates that item associations with the underlying ASA trait were mostly equivalent for men and women, so reporting differences cannot completely explain the differences in associations. The stronger associations observed for men’s ASA with children’s psychopathology should be replicated and explored further in future work.

Parents are a significant part of their children’s environments, and their ASA symptoms may increase children’s risk for separation anxiety via modeling. Parents who become outwardly distressed upon actual or anticipated separation from their children may inadvertently teach children that they are incapable of facing challenges on their own and model temporary separations as distressing and threatening events. Indeed, Bögels and Zigterman (2000) propose that overestimating the danger of being left and underestimating independent functioning may be core cognitive dysfunctions in CSA. Alternatively, ASA may increase the risk for other internalizing disorders like depression via its links with self-criticism (Hock & Lutz, 1998), low self-esteem (McBride & Belsky, 1988), and negative self-representations (Hock & Schirtzinger, 1992).

Parents may also engage in certain parenting behaviors intended to avoid separation. These parenting behaviors may in turn limit children’s opportunities for social interactions and thereby encroach on their abilities to develop a sense of themselves as connected to, but ultimately independent, from their parents. This notion has been discussed in detail in the literature on separation anxiety specifically within the context of the parent–child relationship (e.g. Bartle-Haring, Brucker, & Hock, 2002; Hock & Lutz, 1998). Parents’ ASA may also confer risk for psychiatric outcomes via its consequence in parents. For example, ASA is associated with poor interpersonal functioning, alienation, and aggression (Finsaas & Klein, 2021; Shear et al., 2006); these parents may be more likely to have children with poor interpersonal skills and increased risk for internalizing problems broadly.

Finally, given previous work showing child-to-mother (but not father) effects of anxiety and internalizing symptoms in children and adolescents (Ahmadzadeh et al., 2019; Schulz, Nelemans, Oldehinkel, Meeus, & Branje, 2021; Yirmiya, Motsan, Kanat-Maymon, & Feldman, 2021), it is likely that associations with maternal psychopathology in our study are not solely due to transmission from parent to child. Children with separation anxiety, or other psychiatric, developmental or physical problems, may require more support and attention, which could lead to increases in mothers’ ASA. Alternatively, an external event, like the death of a family member, may lead to disorder onset in parents and children alike.

Finally, the observed associations may also be due to shared genetic liability. Anxiety disorders and related behaviors show moderate heritability across childhood (Boomsma et al., 2005; Trzaskowski, Zavos, Haworth, Plomin, & Eley, 2012). Twin studies of child separation anxiety disorder show that genetic effects explain most individual differences (43% genetic; Scaini, Ogliari, Eley, Zavos, and Battaglia, 2012), but genetically informative studies of anxiety more broadly also provide evidence of environmental effects (Ahmadzadeh et al., 2019; Eley et al., 2015); no studies have used the latter study design for separation anxiety in particular.

Our findings also partially support the validity of ASA as a clinical entity. ASA was associated with offspring internalizing problems, including CSA (Robins & Guze, 1970), but parental ASA was also related to other offspring disorders, providing less support for its distinctness. However, this non-specific patterning of associations was also observed for the much better-established disorder of maternal depression, and thus it may reflect in both cases the limitations of representing psychiatric problems as discrete entities *v*. as specific and sometimes overlapping manifestations of underlying liabilities, a perspective that is gaining in popularity (Kotov et al., 2017), and in line with work showing transmission at the level of internalizing liability (Lahey et al., 2011). Nonetheless, the fact that the associations between ASA and children’s internalizing psychopathology were present over and above other better-established forms of adult psychopathology underscores ASA’s clinical significance; the diagnosis is capturing something not already picked up by other diagnostic categories that relates to children’s psychopathology.

The study findings should also be viewed in light of limitations. Although there is evidence from retrospective reports from adults that CSA precedes ASA (Pini et al., 2010; Shear et al., 2006; Silove et al., 2010b), we do not know whether ASA in our sample was a continuation of CSA or adult-onset. We also cannot distinguish parents’ fear of separation from their children from fear of separating from other attachment figures. Since it is a community sample, we also have relatively low rates of disorders in youth. However, this is tempered by using measures of symptoms alongside diagnoses in structural models. In addition, the reliability of a few of the scales, particularly father’s reports, were in the poor or questionable range, but modeling children’s outcomes as latent factors using multiple sources of information mitigates the impact of suboptimal measurement on associations. The sample is also mostly white, with only a small proportion of Hispanic participants. Although this reflects the demographic makeup of the surrounding community, it limits the generalizability of the results. Moreover, participating fathers were more likely to be married/living with their child’s parent than non-participating fathers, so whether results generalize to fathers who are unmarried or not living with their child’s other parent is not known. Also, there may be unknown biases in samples derived from commercial mailing lists. In addition, while we utilized latent versions of children’s psychopathology, we used a single observed indicator (diagnoses) for parents since we did not have multiple measures of each diagnosis in parents; this may have decreased precision. ASA and other parent psychopathology were also assessed using different types of instruments and covering different assessment periods, which may have impacted predictive power. Subthreshold levels of ASA are captured by the ASA-27 self-report questionnaire, whereas only clinically significant cases of the other forms of psychopathology were captured by the SCID; the higher dimensionality of the ASA variable may have given it more predictive power. At the same time, the SCID, a gold standard tool for assessing psychopathology administered by a trained interviewer, likely produced cleaner representations of psychopathology compared to the self-report ASA-27 instrument, and thus the other parent psychopathology variables may have been more predictive in this way.

In summary, ASA in mothers and fathers is non-specifically related to children’s internalizing problems, including CSA in middle childhood and adolescence, beyond the effects of other common parental mental disorders. Future research should delineate the mechanisms that underlie this intergenerational transmission, particularly parent and child characteristics and parenting behaviors that modify risk.

## Supplementary Material

Supplement

## Figures and Tables

**Table 1.** Reliability statistics for all study measures

	Measure	Variable	Kappa (*n*)	Cronbach’s alpha
Wave 1	SCID	Depressive disorder	0.93 (30)	–
		Anxiety disorder	0.91 (30)	–
		Substance abuse/dependence disorder	1.00 (30)	–
Wave 3	SCID	SCID depressive disorder	0.91 (45)	–
		SCID anxiety disorder	0.73 (45)	–
		Substance abuse/dependence disorder	0.90 (45)	–
	ASA-27	ASA	–	0.91
	K-SADS	Depressive disorder	0.79 (74)	–
		Anxiety disorder	0.68 (74)	–
		Separation anxiety disorder	0.72 (74)	–
	SCARED	Separation anxiety, mother report	–	0.74
		Separation anxiety, father report	–	0.67
		Separation anxiety, child report	–	0.71
		Total, mother report^a^	–	0.89
		Total, father report^a^	–	0.87
		Total, child report^a^	–	0.87
	CDI	Total, mother report^a^	–	0.79
		Total, father report	–	0.76
		Total, child report	–	0.74
Wave 4	K-SADS	Depressive disorder	0.72 (25)	–
		Anxiety disorder	0.75 (25)	–
		Separation anxiety disorder	0.80 (25)	–
	SCARED	Separation anxiety, mother report	–	0.62
		Separation anxiety, father report	–	0.57
		Separation anxiety, child report	–	0.66
		Total, mother report^a^	–	0.90
		Total, father report^a^	–	0.88
		Total, child report^a^	–	0.89
	CDI	Total, mother report^a^	–	0.79
		Total, father report	–	0.80
		Total, child report	–	0.82

a Excludes separation anxiety.

*Notes*: SCID, Structured Clinical Interview for DSM-IV, Non-Patient Version. ASA-27, Adult Separation Anxiety Symptom Questionnaire. K-SADS, The Kiddie Schedule for Affective Disorders and Schizophrenia Present and Lifetime Version. SCARED, Screen for Child Anxiety Related Disorders. CDI, Children’s Depression Inventory.

**Table 2.** Descriptive statistics for children’s anxiety and depressive symptoms from mother, father, and child report

	Wave 3 (Age 9)			Wave 4 (Age 12)		
	*M* (S.D.)	Range	Complete rate	*M* (S.D.)	Range	Complete rate
SCARED: Separation anxiety						

Mother	1.98 (2.38)	0–13	0.94	0.80 (1.38)	0–7	0.90

Father	1.68 (1.98)	0–10	0.82	0.78 (1.28)	0–10	0.73

Child	5.30 (3.28)	0–15	0.93	2.53 (2.24)	0–15	0.91

SCARED: Total excluding separation anxiety						

Mother	5.98 (6.47)	0–43	0.94	7.15 (7.10)	0–40	0.90

Father	5.06 (5.27)	0–31	0.82	6.26 (6.06)	0–39	0.73

Child	14.49 (9.08)	0–53	0.93	14.21 (9.10)	0–52	0.91

CDI						

Mother	7.32 (4.90)	0–29	0.94	7.14 (5.04)	0–27	0.91

Father	7.32 (4.41)	0–23	0.82	7.53 (5.03)	0–24	0.73

Child	4.89 (4.21)	0–22	0.93	4.90 (5.41)	0–33	0.90

*Notes:* For mothers, *n* = 515. For fathers, *n* = 510. For children, *n* = 515. SCARED, Screen for Child Anxiety Related Disorders; CDI, Children’s Depression Inventory.

**Table 3.** Bivariate correlations between parents’ separation anxiety and children’s psychiatric disorders and univariate descriptive statistics for children’s psychiatric disorders

	Separation anxiety		Social phobia		Generalized anxiety		Specific phobia		Depression	
	Age 9	Ages 9–12	Age 9	Ages 9–12	Age 9	Ages 9–12	Age 9	Ages 9–12	Age 9	Ages 9–12
Maternal adult separation anxiety	0.14**	0.11*	0.00	0.04	0.16***	0.12**	0.03	0.01	0.14**	0.01

Paternal adult separation anxiety	0.03	0.19***	0.00	0.01	0.05	0.00	0.08^^^	0.01	0.02	−0.03

Proportion (*n*)	0.06 (28)	0.03 (16)	0.04 (18)	0.05 (23)	0.04 (21)	0.05 (22)	0.11 (56)	0.09 (45)	0.02 (11)	0.06 (29)

Notes:

*** *p* < 0.001;

** *p* < 0.01;

* *p* < 0.05;

^ *p* < 0.10.

Age 9 = wave 3 assessment, which covered child’s lifetime through age 9. Ages 9-12 = wave 5 assessment, which was done when children were 12 years old and covered the period since the age 9 assessment. Parents’ adult separation anxiety assessed at wave 3. Correlations with agoraphobia and panic disorder are not reported here because of low counts (<1%) but these variables are included in structural models.

**Table 4.** Model fit statistics

	CFI	TLI	RMSEA (90% CI)	χ^2^	*df*	*p*	*n*
Maternal							
Concurrent	0.945	0.902	0.037 (0.028–0.045)	213.466	129	<0.001	488
Prospective	0.938	0.890	0.043 (0.035–0.051)	242.09	129	<0.001	475
Paternal							
Concurrent	0.950	0.910	0.033 (0.024–0.042)	198.85	129	0.0001	484
Prospective	0.929	0.873	0.044 (0.036–0.052)	2456.25	129	<0.001	470

*Notes:* CFI, comparative fit index; TLI, Tucker-Lewis Index; RMSEA, root mean square error of approximation.
