# Supplementary material for: Is adult separation anxiety associated with offspring risk for internalizing psychiatric problems?
Source: Psychol Med. 2022 Jan 26;53(7):3168–77. doi: 10.1017/S0033291721005249 (PMC10187056; doi:10.1017/S0033291721005249)
Supplement: Supplementary file 1 [file S0033291721005249sup001.docx]

Supplemental Table 1

*Select item descriptions from Adult Separation Anxiety Questionnaire (ASA-27)*

| Item |  | DSM-IV |
| --- | --- | --- |
| 2^a^ | Difficulty staying away from home for hours | A-4 |
| 5^a^ | Nightmares about separation from attachments | A-7 |
| 12^a^ | Difficulty sleeping alone at night | A-6 |
| 14^a^ | Very distressed thinking about being away from attachments | A-1 |
| 16^a^ | Worries about attachments coming to serious harm | A-2 |
| 20^a^ | Avoids being home alone when attachments are out | A-5 |
| 23 | Afraid would not be able to cope if attachments left | -- |
| 24^a^ | Panic symptoms when separated from attachments | A-8 |
| 25^a^ | Worrying about events causing separation from attachments | A-3 |
| 27 | Worries relationship so close it causes others problems | -- |

Notes: ^a^ Items based on DSM-IV criteria (Manicavasagar, Silove, Curtis, & Wagner, 2000); the particular DSM-IV criteria was determined by the authors of the current study.

Supplemental Table 2

*Demographic variable descriptions*

| Participant | Variable | Variable type | Variable description |
| --- | --- | --- | --- |
| Mother | Age | Continuous | Age in years assessed at age 9 visit |
|  | Education | Binary | College graduate = 1 |
|  | Relationship status | Binary | Married to and/or living with child’s biological father = 1 |
| Father | Age | Continuous | Age in years assessed at age 9 visit |
|  | Education | Binary | College graduate = 1 |
|  | Relationship status | Binary | Married to and/or living with child’s biological father = 1 |
| Child | Age 9 | Continuous | Age in years assessed at age 9 visit |
|  | Age 12 | Continuous | Age in years assessed at age 12 visit |
|  | Gender | Binary | Female = 1 |
|  | Ethnicity | Binary | Hispanic or Latino = 1 |
|  | Race | Categorical | White; Black; Asian; Pacific Islander; Native American; other |

Supplemental Table 3

*Missing data summary*

|  |  | Descriptive statistics | Significance tests |
| --- | --- | --- | --- |
| Mother | Married/living with biological father^1^ | 413/490 (84.3%) v. 1/3 (33.3%) | *χ*^2^(1, *N*=493)=2.59, *p=*.11 |
|  | College graduate^1^ | 277/492 (56.4%) v. 1/3 (33.3%) | *χ*^2^(1, *N*=492)=.05, *p=*.83 |
|  | Age | *M=*41.8, *SD=*4.83 v. *M=*41.1, *SD=*2.04 | *t*(489)=-0.21, *p=*.84 |
|  |  |  |  |
| Father | Married/living with biological mother^1^ | 411/486 (84.6%) v. 4/7 (42.9%) | *χ*^2^(1, *N*=493)=6.09, *p=*.01 |
|  | College graduate^1^ | 216/487 (44.4%) v. 2/5 (40.0%) | *χ*^2^(1, *N*=492)=0.00, *p=*1.00 |
|  | Age | *M=*44.0, *SD=*5.85 v. *M=*44.1, *SD=*7.86 | *t*(488)=0.03, *p=*.97 |
|  |  |  |  |
| Child | Hispanic-Latino | 46/515 (12.4%) v. 11/94 (11.7%) | *χ*^2^(1, *N*=609)=0.00, *p=*.98 |
|  | Age at age 9 visit | *All cases that had with data on the age variable at this visit were included in the study sample* |  |
|  | Age at age 12 visit | *M=*12.7, *SD=*0.46 v. *M=*13.2, *SD=*NA (only one case in this group) | *t*(474)=1.03, *p=*.30 |
|  | Gender | 239/515 (46.4%) v. 38/94 (40.4%) | *χ*^2^(1, *N*=609)=0.92, *p=*.34 |

^1^Due to small cell size, Chi-squared approximation may be incorrect.
